# Platelet Mechanobiology Inspired Microdevices: From Hematological Function Tests to Disease and Drug Screening

**DOI:** 10.3389/fphar.2021.779753

**Published:** 2022-01-20

**Authors:** Yingqi Zhang, Fengtao Jiang, Yunfeng Chen, Lining Arnold Ju

**Affiliations:** ^1^ School of Biomedical Engineering, Faculty of Engineering, The University of Sydney, Sydney, NSW, Australia; ^2^ Charles Perkins Centre, The University of Sydney, Camperdown, NSW, Australia; ^3^ Heart Research Institute, Newtown, NSW, Australia; ^4^ The Department of Biochemistry and Molecular Biology, The University of Texas Medical Branch, Galveston, TX, United States; ^5^ The Department of Pathology, The University of Texas Medical Branch, Galveston, TX, United States

**Keywords:** microfluidics, thrombosis, platelet, von Willebrand disease, mechanobiology, clopidogrel, aspirin, COVID-19

## Abstract

Platelet function tests are essential to profile platelet dysfunction and dysregulation in hemostasis and thrombosis. Clinically they provide critical guidance to the patient management and therapeutic evaluation. Recently, the biomechanical effects induced by hemodynamic and contractile forces on platelet functions attracted increasing attention. Unfortunately, the existing platelet function tests on the market do not sufficiently incorporate the topical platelet mechanobiology at play. Besides, they are often expensive and bulky systems that require large sample volumes and long processing time. To this end, numerous novel microfluidic technologies emerge to mimic vascular anatomies, incorporate hemodynamic parameters and recapitulate platelet mechanobiology. These miniaturized and cost-efficient microfluidic devices shed light on high-throughput, rapid and scalable platelet function testing, hematological disorder profiling and antiplatelet drug screening. Moreover, the existing antiplatelet drugs often have suboptimal efficacy while incurring several adverse bleeding side effects on certain individuals. Encouraged by a few microfluidic systems that are successfully commercialized and applied to clinical practices, the microfluidics that incorporate platelet mechanobiology hold great potential as handy, efficient, and inexpensive point-of-care tools for patient monitoring and therapeutic evaluation. Hereby, we first summarize the conventional and commercially available platelet function tests. Then we highlight the recent advances of platelet mechanobiology inspired microfluidic technologies. Last but not least, we discuss their future potential of microfluidics as point-of-care tools for platelet function test and antiplatelet drug screening.

## Introduction

In blood circulation, anucleate platelets are the smallest cells that play a central role in hemostasis (hemorrhage arrest upon vascular breach) and thrombosis (vessel occlusion with cessation of blood flow leading to tissue injury) (Rasche, 2001; Ruggeri, 2002; Colman, 2006). In line with the Virchow’s triad (Bagot and Arya, 2008), platelet adhesion, activation and aggregation are significantly influenced by hemodynamic factors such as shear rate and shear stress (Yin et al., 2011; Sheriff et al., 2013). In pathologically relevant vascular anatomies including stenoses, aneurysms and bifurcations, platelet prothrombotic behaviors are further exacerbated by shear gradient (Nesbitt et al., 2009; Westein et al., 2013; Zhang and Neelamegham, 2017), vorticity (Varble et al., 2017), or turbulence (Nesbitt et al., 2009; Ha et al., 2018). At the molecular scale, increasing evidences suggest that platelets can undergo mechanosensing upon receiving these hemodynamic stimuli. Key players in such mechanosensing processes include von Willebrand factors (VWF) (Savage et al., 1996; Fu et al., 2017), fibrinogen (Butera and Hogg, 2020; Peshkova et al., 2020), mechanoreceptors including glycoprotein Ib (GPIb) (Chen et al., 2016; Ju et al., 2016) and glycoprotein IIb/IIIa (GPIIb/IIIa or integrin α_IIb_β_3_) (Nesbitt et al., 2009; Chen Y et al., 2019), and mechanosensitive ion channels (Abbonante et al., 2017; Ilkan et al., 2017; Liu et al., 2021). Further, activated platelets generate contractile forces to stabilize and consolidate the thrombus (Osdoit and Rosa, 2001; Ono et al., 2008; Hansen et al., 2018). Abnormal platelet function can cause thrombosis (Chen and Ju, 2020), bleeding disorders (Castaman et al., 1997); Cines and Bussel, 2021; Lee et al., 2021; Pavord et al., 2021) and autoimmune diseases (Zoller et al., 2012). Diabetes, obesity and other metabolic syndromes are well known to be associated with platelet hyperactive functions and exhibit prothrombotic phenotypes (Podrez et al., 2007; Santilli et al., 2012; Ju et al., 2018). More recently, COVID-19 severe symptoms and thrombotic complications are demonstrated to associate with platelet dysfunctions (Manne et al., 2020; Koupenova et al., 2021).

Over the past decades, multiple platelet function tests—mainly grouped into biomarker-based assays, aggregometry and biomechanical-based assays—have been commercialized and standardized for diagnosis and monitoring of platelet (dys)function in clinical pathology laboratories and intensive care units (Yarovoi et al., 2003; Nesbitt et al., 2006; Furie et al., 2021). However, due to the requirement of large sample volumes and long processing time, these expensive and bulky techniques often have restricted application. More importantly, the hemodynamic microenvironment and platelet mechanobiology at play are insufficiently incorporated in these tests. While the existing biomechanical assays have incorporated the flow effect and viscoelasticity of platelet thrombi, they often have fixed physical constants and black boxes for external manipulation. To this end, more handy, efficient, and inexpensive point-of-care tools that can incorporate platelet mechanobiology promise more comprehensive and profound assessment of platelet function and the related hematological disorders (Paniccia et al., 2015).

With respect to antiplatelet medications, aspirin and triflusal (TxA2 generation blockers), clopidogrel, ticagrelor, ticlopidine and prasugrel (adenosine diphosphate (ADP) receptor P2Y_12_ blockers), dipyridamole and cilostazol (phosphodiesterase inhibitors), vorapaxar (PAR-1 antagonist), and warfarin (vitamin K antagonists) are the standards of care that target platelet functions for antithrombotic therapies (Dlott et al., 2014; Thachil, 2016; McFadyen et al., 2018). However, longstanding limitation of these agents is their inability to differentiate between hemostasis and thrombosis. Adverse side effects associated with these antiplatelet therapies can appear, including the increased risk of dose-dependent bleeding (by prasugrel and ticagrelor), thrombocytopenia (by heparin, prasugrel, ticlopidine), hypersensitivity (by clopidogrel, prasugrel, ticagrelor), acute kidney injury (by aspirin) and hypotension (by dipyridamole) (Walenga et al., 2012; McFadyen et al., 2018). Therefore, the dose and combination of antiplatelet prescription needs to be tailored carefully upon individuals (Koenig-Oberhuber and Filipovic, 2016). There is a strong driver for rapid, quantitative and accurate analytical tools that have utility with respect to patient-specific antiplatelet therapies, in other words, more effective antiplatelet precision medicine.

With recent advance of microfabrication technologies, a variety of microfluidic approaches emerge to mimic vascular anatomies, reconstitute hemodynamic factors and recapitulate platelet mechanobiology underlying hematological processes (Tovar-Lopez et al., 2010; Tsai et al., 2012; Zilberman-Rudenko et al., 2017; Koupenova et al., 2018; Ting et al., 2019). Whilst clinical translation remains a pertinent issue, the miniaturized and cost-efficient microfluidic devices are the complementary avenues that allow rapid and high-throughput platelet function testing and antiplatelet drug screening. To date, several point-of-care microfluidic systems have gained FDA approvals for clot viscoelasticity assay (TEG^®^ 6s system), blood chemistry analysis (sodium, potassium, chloride, glucose, hematocrit, gases) and immune hematology tests (analytes concentration) (Chin et al., 2012; Sharma et al., 2015).

Hereby, we summarize the existing platelet function tests in the clinical domain, discuss their limitations, then review emerging microfluidic devices inspired by platelet mechanobiology and discuss their future point-of-care potentials.

## Commercially Available Platelet Function Tests and Their Clinical Usage

Bleeding time (BT) evaluation appeared as the earliest platelet function test in clinical use (Duke, 1983). Serving as an *in vivo* testing, BT is invasive and has low reproducibility and specificity in the routine monitoring of antiplatelet therapies (Jakubowski et al., 2007). Recently, noninvasive and simpler *in vitro* platelet function tests become commercially available. We list these existing techniques in Table 1—A summary of the standardized platelet function tests, which could be broadly categorized into the following three groups:

1) Biomarker-based assessment. Platelet functional status are often depicted by their activation marker expression and metabolite secretion. Flow cytometry is commonly used to not only quantify platelet receptor expression such as GPIb (Adelman et al., 1985), but also depict platelet activation status via PAC-1 antibody binding (GPIIb/IIIa activation) (Ginsberg et al., 1990; Ju et al., 2018), P-selectin expression (α-granule secretion) (Kehrel and Brodde, 2013), annexin A5 binding (phosphatidylserine exposure) (Reddy et al., 2018), and vasodilator-stimulated phosphoprotein-phosphorylation (P2Y_12_ activation) (Hezard et al., 2010; Williams et al., 2010; Harrison and Keeling, 2012; Kehrel and Brodde, 2013; Dahlen et al., 2013; Harrison and Lordkipanidze, 2013; Paniccia et al., 2015; Reddy et al., 2018). The related deficiency and mutation can be quickly identified by flow cytometry and linked to platelet disorders such as Bernard–Soulier syndrome (BSS) (GPIb), Glanzmann’s thrombasthenia (GT) (GPIIb/IIIa) and platelet storage pool diseases such as gray platelet syndrome (α-granule).

**TABLE 1:** A summary of conventional assays and the novel microfluidic devices for platelet function analysis and antiplatelet drug screening.

Analysis	Device	Measurement	Clinical implication	Pharmacologic monitor	Advantages	Limitations	References
**Commercial devices**							
Biomarker based	Flow cytometry, ELISA	Platelet activation markers quantification	BSS/GT/HIT/Scott syndrome	Aspirin, P2Y_12_ antagonists, heparin	Small volume/independent of platelet count	Expensive/specialized training	Muir et al. (2009), Hezard et al. (2010), Williams et al. (2010), Pakala and Waksman (2011), Harrison and Keeling (2012); Kehrel and Brodde (2013); Dahlen et al. (2013), Gremmel et al. (2013), Harrison and Lordkipanidze (2013), Guéry et al. (2018)
Aggregom-etry	Light Transmission Aggregometry (VerifyNow^®^, AggRAM™, APACT 4004^®^, PAP-8E^®^)	Optical density	ADP accumulation defect/BSS/Type 2B VWD/GT	Aspirin, P2Y_12_ and GPIIb/IIIa antagonists	Flexible/gold standard	Sample processing/large sample volumes/lack HCT consideration/not sensitive to acquired platelet defects	Hayward et al. (2009), Morel-Kopp et al. (2010), Sibbing et al. (2010), Solomon et al. (2010), Bolliger et al. (2012a), Tantry et al. (2013), Paniccia et al. (2015), Opheim et al. (2019), Alessi et al. (2020), Le Blanc et al. (2020)
	Multiple Electrode Aggregometry (Multiplate^®^, Chrono-Log^®^)	Electrical impedance	Storage pool disease/GT/VWD/COD/HIT	Aspirin, P2Y_12_ and GPIIb/IIIa antagonists	Simple/small volume/flexible	Limited HCT and platelet count range/bulky/insensitive to TRAP-induced platelet aggregation	
Biomechan-ical based	Shear flow-based assays (PFA-100/200^®^, PlaCor PRT^®^)	Occlusion time	Type 2 VWD/BSS/GT	Aspirin, P2Y_12_ antagonists	Rapid/small volume/simple/sensitive to severe platelet defects	Insensitive to mild platelet disorders/platelet count and HCT dependent/irrelevant to stenotic thrombosis	Harrison et al. (2002), Harrison et al. (2011), Johnson et al. (2012), Gorog and Jeong (2015)
	Cone and Plate (Let) Analyzer (Impact-R^®^)	Surface coverage and aggregation size	Type 3 VWD/GT/Afibrinogenemia	Aspirin, GPIIb/IIIa antagonists, ADP antagonists	Automated/simple/rapid/small volume/	Expensive/specialized training/lack clinical studies	Savion and Varon (2006), Anand et al. (2007), Shenkman et al. (2008), Paniccia et al. (2015)
	Thromboelasto-graphy assay (TEG^®^, ROTEM^®^, Sonoclot^®^)	Clot viscoelasticity upon torque application	ACT/PPH	Heparin, aprotinin, aspirin, GPIIb/IIIa antagonists, ADP antagonists	Complete clot profile	Interlaboratory variation/time-consuming/limited platelet and HCT count range/lack clinical study/expensive	Paniccia et al. (2015), Mitrovic et al. (2021), Moore et al. (2021)
	Thromboelast-ography assay (TEG^®^ 6s, Quantra^®^)	Clot viscoelasticity upon resonance application	Trauma and cardiac surgery	P2Y_12_ and GPIIb/IIIa antagonists	High precision/fully automated/portable/multi-channel/reduced blood volume	Lack clinical study/expensive	(Ferrante et al., 2016; Dias et al., 2020; Lloyd-Donald et al., 2020)
**Microfluidic platforms**							
Shear dependent platelet function test	Straight	Platelet adhesion	-	COX-1, P2Y1 and P2Y_12_ antagonists	Controlled flow rate/temporal and spatial observation	-	Li et al. (2017)
	Stenosis	Clotting time	HPS/Sepsis/SCA	COX, P2Y_12_, GPIIb/IIIa antagonists (aspirin, clopidogrel, abciximab), Heparin	High dynamic range/real-time monitoring and quantification	Non instantaneous and continuous (<20min) monitoring	Jain et al. (2016b)
		Platelet aggregation surface and size	Borderline type 1 VWD; Type 2/3 VWD	T_X_A2, P2Y_12_ and P2Y1 antagonists (indomethacin, 2-E11MeSAMP, MRS2179)	Real-time monitoring/small volume/sensitive to low platelet count	Strict to ULVWF involved aggregation	Brazilek et al. (2017)
Contractile force analysis	Micropatterns	Microdot area and displacement	WAS/MYH9RD	–	Single cell resolution/modulable substrate properties/high throughput	Cannot detect low contraction	Myers et al. (2017)
	Microposts	Micropillar deflection	TIC/cardiology patient on aspirin medication	P2Y_12_, GPIb/V/IX, GPIIb/IIIa antagonists (2-MeSAMP, AK2, c7E3)	No additional agonist required/No sample preparation/sensitive	–	Ting et al. (2013), Ting et al. (2019), Miles et al. (2021)
			Type 2A VWD	GPIbα, GPIIb/IIIa antagonists (HIP1, abciximab)	Real-time/medium throughput/clot stiffness measurement	–	Chen Z et al. (2019)
Integrated drug screening system	SpearChip	Platelet adhesion	–	GPIIb/IIIa and P2Y_12_ antagonists (abciximab, clopidogrel, prasugrel, ticagrelor, cangrelor)	Self-powered/no dead volume/reproducible	Flow controlled by chip design	Jose et al. (2016)
	Micropump-Mixer	Thrombus volume	–	PI3K inhibitors (AS2524224, TGX221, LY294002, Wortmannin)	High integration/high throughput/automated/short incubation time/small dead volumes	–	Szydzik et al. (2019)

BSS, Bernard–Soulier syndrome; GT, Glanzmann’s thrombasthenia; HIT, Heparin-induced thrombocytopenia; COD, Cyclooxygenase deficiency; ATC, Acute trauma coagulopathy; PPH, Postpartum hemorrhage; HPS, Hermansky–Pudlak syndrome; SCA, sickle cell anemia; WAS, Wiskott–Aldrich; MYH9RD, MYH9-related disorders; TIC, Trauma-induced coagulopathy; VWD, von Willebrand disease; HCT: hematocrit.

Besides, measurement of thromboxane metabolites (T_X_A2) allows evaluation of platelet activation using ligand-binding assays such as radioimmunoassay (Rogasi et al., 1988), immunoradiometric assays (Shen and Tai, 1986), or enzyme-linked immunoassays (ELISA) (Muir et al., 2009; Gremmel et al., 2013). The LabCorp Serotonin Release Assay is considered as the gold standard for diagnosing heparin-induced thrombocytopenia (HIT) (Guéry et al., 2018). While these biomarker-based assays use small sample volumes and can be independent on platelet counts, they are generally time-consuming, expensive, and require specialized operators and core facilities.

2) Aggregometry assays. There are mainly two types of platelet aggregation measurement—Light Transmission Aggregometry (LTA) (Alessi et al., 2020) and Multiple Electrode Aggregometry (MEA) (Opheim et al., 2019). LTA is the gold standard platelet function test that observes the increase of light transmission through the platelet-rich plasma (PRP) or washed platelet sample due to the convergence of individual platelets into aggregates; whereas MEA evaluates the electrical impedance proportional to platelet aggregation (Paniccia et al., 2015). There are a few LTA (VerifyNow^®^, AggRAM^®^, APACT 4004^®^, PAP-8E^®^) and MEA (Multiplate^®^, Chrono-Log^®^) currently available for disease screening (storage pool disease, HIT, GT, BSS, von Willebrand disease (VWD), ADP accumulation defects or cyclooxygenase deficiency) (Morel-Kopp et al., 2010; Bolliger et al., 2012a; Tantry et al., 2013; Opheim et al., 2019; Alessi et al., 2020) and antiplatelet drug monitoring (aspirin, P2Y_12_ and GPIIb/IIIa antagonists) (Sibbing et al., 2010; Solomon et al., 2010; Le Blanc et al., 2020).

Nevertheless, these aggregometries present a few limitations. For example, LTA has less pronounced sensitivity to acquired platelet defects (Hayward et al., 2009), and MEA has not yet been able to distinguish those not responding to antiplatelet drugs and at risk of major adverse cardiovascular events (Paniccia et al., 2015).

3) Biomechanical platelet function assays. Biomechanical tests can be broadly grouped into shear flow -based assays [PFA-100/200^®^, PlaCor PRT^®^ (Johnson et al., 2012)], Cone and Plate(let) Analyzer (CPA) [Impact-R^®^ (Savion and Varon, 2006)], and thromboelastography assays [TEG^®^ (Korpallova et al., 2018), ROTEM^®^ (Bolliger et al., 2012b; Zaky, 2017), Sonoclot^®^ (Ganter and Hofer, 2008), TEG^®^ 6s (Lloyd-Donald et al., 2020), Quantra^®^ system (Ferrante et al., 2016)].

PFA-100/200^®^ and PlaCor PRT^®^ measure the occlusion time after exposing platelets to a constant shear rate at 5,000 s^−1^ and 1,500 s^−1^, respectively (Gorog and Jeong, 2015). PFA-100/200^®^ immobilizes exogenous antagonists on a solid cartridge for platelet activation while PlaCor PRT^®^ activates platelets by inducing high shear in a narrowed aperture with a spring (Godino et al., 2014). These shear flow-based assays can distinguish type 2 VWD, BSS and GT but not mild platelet defects such as Hermansky–Pudlak syndrome (HPS), storage pool and release defects, type 1 VWD and macrothrombocytopenia (Harrison et al., 2002; Harrison et al., 2011). CPA assesses adhering platelets subjected to shear forces imposed by the spinning cone on a plate. This system is useful to monitor type 3 VWD and examine dual antiplatelet drug efficiency but need to be further verified for inherited and acquired platelet dysfunction diagnosis (Savion and Varon, 2006; Anand et al., 2007; Shenkman et al., 2008; Paniccia et al., 2015).

Not specific to platelet function analysis, the thromboelastography is a global hemostatic function assay that evaluates viscoelastic variations of clot retraction (both platelet aggregation and fibrin polymerization) to diagnose coagulopathies (e.g., hypofibrinogenemia, platelet dysfunction) (Da Luz et al., 2014), predict bleeding risks (Stravitz, 2012), and determine need for transfusion (Paniccia et al., 2015). Nevertheless, thromboelastography assays may have two major limitations: 1) baseline measurements prior to treatment are required for reference; and 2) single platelet mechanosensing phenotypes are masked by high amounts of thrombin generated and the subsequent clot retraction (Moore et al., 2021).

## Novel Microfluidic Approaches for Platelet Function Assessment and Anti-Platelet Drug Screening

While the aforementioned commercial platelet function tests have been broadly used in clinical practices, emerging microfluidic approaches demonstrate several advantages:

1) Microfluidics can emulate physiologically relevant vascular anatomies. Conventional methods such as PFA-100/200^®^ do not capture the geometric characteristics of the vessel, preventing the mimicking of platelet responses to their microenvironment. In contrast, latest soft lithography enables multifaceted, high-fidelity and customized microfluidic designs to imitate various vascular anatomies, such as straight (Jain et al., 2016b; Li et al., 2017; Zhang and Neelamegham, 2017; Albers et al., 2019; Dupuy et al., 2021), bifurcated (Tsai et al., 2012), stenosed (Tovar-Lopez et al., 2010; Jain et al., 2016b) and net (Zilberman-Rudenko et al., 2017; Zhang et al., 2021) channels.
2) Microfluidics can recapitulate hemodynamic microenvironment. It is almost impossible for conventional platelet function tests to encapsulate the rheological parameters experienced by the platelets. Even for the PFA-100/200^®^, pre-exposure to exogenous agonists hinders them to fully recapitulate the synergistic effects of chemical (released endogenously by activated platelets) and mechanical factors (shear stress, shear gradients, vorticity) (Panzer and Jilma, 2011). By mediating the geometries and flow input, microfluidic approaches have great control on the hemodynamic parameters which can be predicted when combined with computational fluid dynamics (CFD) simulation (Zhao et al., 2021).
3) Microfluidics have higher sensitivity in detecting certain platelet mechanobiology relevant disorders. While sometimes not readily and obviously diagnosed by the existing platelet function tests, HPS, VWD, Wiskott–Aldrich syndrome and MYH9-related disorders can be effectively detected with a few platelet mechanobiology inspired microfluidics (Jain et al., 2016b; Brazilek et al., 2017; Myers et al., 2017; Chen Z et al., 2019; Ting et al., 2019).
4) Microfluidics present better biomimetic performance. Endothelialized microfluidics are rapidly evolving as humanized screening platforms (Albers et al., 2019; Jenny et al., 2020; Dupuy et al., 2021). Increasing evidences demonstrate their great potential in simulating the interplays between platelets and circulatory systems (Zhang et al., 2021).
5) Existing platelet function tests are bulky, expensive, and require specialized operators and a large volume of blood samples. In sharp contrast, microfluidic devices are cheap in terms of both materials and fabrication process. These miniaturized microdevices are in sub-millimeter dimension (Chiu et al., 2017), which only require a small volume of blood sample in the scale of microliters (Convery and Gadegaard, 2019) to render reliable results. More recently, the integrated microfluidics with micro-pumps and mixers enable high-throughput, automated disease and drug screening in much shorter turnaround time (Mayr and Bojanic, 2009; Szydzik et al., 2019).

In the following three subsections and Table 1, we summarized novel microfluidic platforms as the potential point-of-care tests of platelet function and antiplatelet drug screening.

### Shear Dependent Platelet Mechanobiology Inspired Microfluidics

A range of microfluidic devices that recapitulate physiological and pathological hemodynamic microenvironment have been employed for platelet thrombosis and hematological studies (Gutierrez et al., 2008; Tovar-Lopez et al., 2010; Conant et al., 2011; Costa et al., 2017; Menon et al., 2020). The newly obtained platelet mechanobiology have further inspired novel microfluidic designs for diagnosis of platelet function disorders and patient profiling (Myers et al., 2017; Chen Z et al., 2019).

The earlier study of the shear dependent platelet mechanobiology utilized the simple straight channel with rectangular cross-section which can be easily fabricated by standard soft lithography. Gutierrez et al. (2008) developed two PDMS microfluidics devices with small cross section areas, thereby reducing the blood volume required (<100 µl per assay) under a range of shear rates (13—1,310 s^−1^). Further, Li et al. (2017) applied an eight-channel microfluidic device coated with collagen to test the efficacy, dosage response, and combined antiplatelet therapeutic outcomes. The effectiveness of antiplatelet drugs (P2Y, COX-1 and kinase inhibitors) was tested on whole blood obtained from healthy individuals under the shear rates of 200 s^−1^ and 1,000 s^−1^. Similarly, Rossi and Diamond (2020) designed an injection-molded microfluidic device with a collagen/tissue factor-printed surface to evaluate the dose response of anticoagulants (dabigatran, rivaroxaban, apixaban).

**FIGURE 1:**
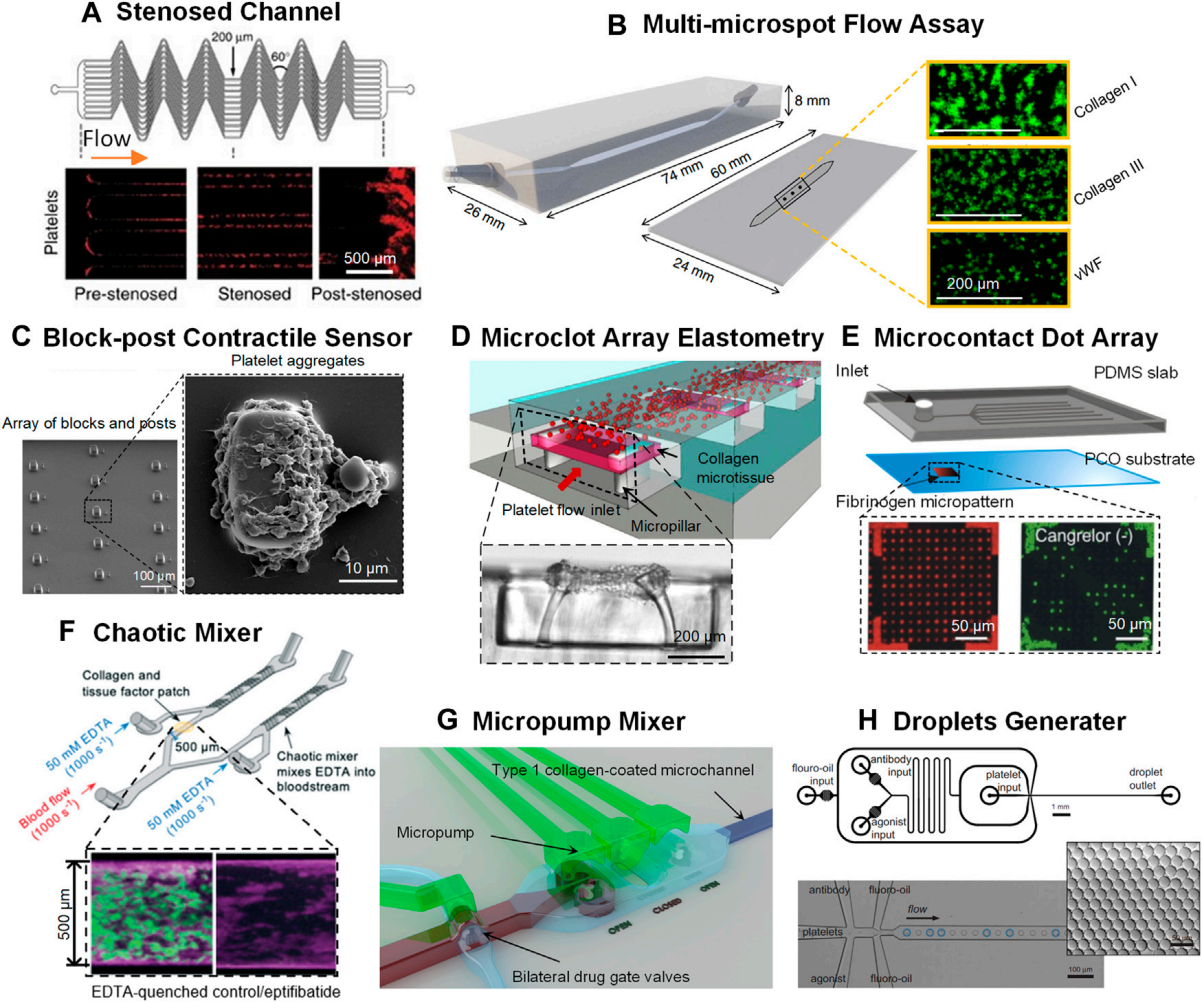
Novel microfluidic platforms as point-of-care test of platelet function and anti-platelet drug screening. **(A)** A network of parallel stenosed microchannels which contain multiple regions of pre-stenosis, stenosis and post-stenosis from Jain et al. (2016a). **(B)** A multi-microspot microfluidic flow system from de Witt et al. (2014); Zoom-in: thrombi formed on the collagen/VWF microspot surfaces. Scale bar = 200 μm. **(C)** Block-post contractile sensor from Ting et al. (2013), Ting et al. (2019); Left: SEM micrograph of an array of blocks and posts. Scale bar = 100 μm; Right: SEM micrograph of a platelet aggregate formed after 45 s at 8,000 s^−1^. Scale bar = 10 μm. **(D)** A microclot array elastometry system from Chen Z et al. (2019); Top: An array of exposed collagen microtissues capturing the flowing platelets to form individual microclots; Bottom: Sideview of a microtissue after platelet-mediated contraction. Scale bar = 200 μm. **(E)** The microcontact printed dot arrays from Jose et al. (2016); Top: Schematic of the microfluidic assembly; Bottom: Fluorescence images of 6-μm Cy-3-labeled fibrinogen-dot arrays (red dots) and of FITC-labeled platelets (green dots) adhering to the fibrinogen dot arrays. Scale bar = 50 μm. **(F)** A chaotic mixer from Berry et al. (2021); Top: Schematic of the chaotic mixer; Bottom: Confocal images of platelets (and leukocytes) and fibrin in EDTA-quenched channel (left) and the eptifibatide channel (right). **(G)** An active micropump mixer with micropump valve chambers and pneumatic actuation chambers from Szydzik et al. (2019). **(H)** Droplet microfluidics from Jongen et al. (2020); Top: Schematic of the droplet generator design. Scale bar = 1 mm. Bottom: Monodisperse droplets encapsulating platelets. Scale bar = 100 μm. Zoom-in: droplet monodispersity is indicated by hexagonal packaging. Scale bar = 50 μm.

With recent microfabrication advancement, pathological microvascular geometries were incorporated into microfluidic channels to recapitulate flow disturbance and examine shear gradient effects on platelet thrombotic functions (Tovar-Lopez et al., 2010). Notably, Jain *et al.* developed a stenosed arteriole-mimicking microfluidics that consists of three regions: 1) pre-stenosed region with sudden fluid acceleration; 2) stenosed region with uniform shear; 3) post-stenosed region with abrupt flow deceleration (Figure 1A) (Jain et al., 2016a). Remarkably, such microdevice was able to measure hemostatic defects of patients with HPS. The platelet defect in HPS patients is not commonly detectable by conventional PFA-100 and bleeding time assays (Harrison et al., 2011). Additionally, Brazilek et al. (2017) presented a micro-contraction device where 80° double-stenosed test segments were designed. This stenosis microchannel not only can detect the reduction of biomechanical platelet aggregation as implicated in patients with type 1, 2 and 3 VWD, but also can distinguish the borderline type 1 VWD from the severe one. Interestingly, this microdevice gave better diagnostic outcomes in type 1 VWD patients than PFA-100 assay. One of the reason may be that the defective phenotypes of shear-dependent VWF–platelet mechanobiology is masked by the potent platelet pre-activation in the exogenous agonists coated cartridges in PFA-100 (Ardillon et al., 2015; Favaloro, 2015).

Other complexed vasculature mimicking systems have been developed, such as bifurcation microchannels (Tsai et al., 2012; Mao et al., 2021), vascular inflammatory model (Johnston et al., 2020) and bleeding model (Hu et al., 2021). These innovative microfluidic platforms are capable of recapitulating not only the pathological shear but also the vascular biological functions for thrombosis, hemostasis and thromboinflammation studies and platelet function tests and drug screening. Moreover, de Wett *et al.* introduced a multi-microspot microfluidic flow system made of 52 platelet adhesive proteins and eight output parameters to characterize thrombus formation under wall shear rates at 150 s^−1^ and 1,600 s^−1^ (Figure 1B) (de Witt et al., 2014). Strikingly, the system has been applied to reveal abnormal thrombus formation in patients with severe combined immune deficiency, GT, HPS, May–Hegglin anomaly or gray platelet syndrome.

### Microfluidic Devices That Gauge Platelet Contractile Force

Decreased platelet contractility is associated with the abnormal mechanics of blood clots. Several studies have confirmed the high relevance of platelet contractile forces to platelet aggregation and the subsequent hemostasis (Li and Li, 2006; Muthard and Diamond, 2012; Muthard and Diamond, 2013; Ting et al., 2013; Chen Z et al., 2019; van Rooij et al., 2020). Lam et al. (2011) customized a side-view atomic force microscope (AFM) to measure the contractile force of a single platelet encapsulated between the fibrinogen-coated cantilever and surface. Moreover, DNA-based tension probes are emerging as novel nanotechnology to measure platelet traction force by quantifying the threshold force required to unfold the immobilized DNA hairpins that links to platelet integrin receptors (Dutta et al., 2018; Zhang et al., 2018; Zhao et al., 2019).

While the existing AFM and DNA tension probes do not directly recapitulate hemodynamic effects, Hanke et al. (2019) introduced a microfluidic chamber integrated with traction force microscopy to quantify platelet contraction when exposed to shear. Specifically, a polyacrylamide hydrogel encapsulated with fluorescent beads was attached to the bottom of the microfluidic chamber. By measuring the displacement of substrates indicated by fluorescence beads, one can measure the platelet contractile forces under shear.

Further, Myers et al. (2017) developed a high-throughput platelet contraction cytometry that is capable of evaluating platelet contractile forces at single-cell resolution. This microfluidic device is composed of three layers: 1) a bottom cover slip; 2) a laser cut PDMS gasket filled with fibrinogen microdots-patterned hydrogel; and 3) a microfluidic flow chamber with inlet and outlet. In this model, single platelet contractility is directly associated with the area and displacement of fibrinogen microdot. Notably, patients suffering from Wiskott–Aldrich syndrome or MYH9-related disorders were identified to lack highly contractile platelets (Myers et al., 2017) and clot contraction (Godwin and Ginsburg, 1974; Shcherbina et al., 2010). However, one limitation of this device is that low contractile platelets are not detectable and therefore optimization is needed for more profound platelet contractility analysis.

In addition to single-cell level measurement, Ting *et al.* examined platelet contractility based on platelet aggregation using a microfluidic device with an array of rectangular micro-blocks paired with flexible microposts (Figure 1C) (Ting et al., 2013; Ting et al., 2019). Platelet contractile forces can be quantified based on the deflections of the microposts. Measurement of platelet contractile forces could help differentiate healthy individuals from patient-specific conditions, for example, cardiology patients on antiplatelet (aspirin) medications or trauma-induced coagulopathy patients at risk of bleeding. Besides, Chen *et al.* introduced a microclot array elastometry system consisting of three layers: 1) a microchannel on top; 2) collagen microtissues formed on PDMS micropillars in the intermediate layer; 3) stretchable silicone membrane on the bottom layer to allow micropillar deflection for clot contraction measurement (Figure 1D) (Chen Z et al., 2019). Such system has been used to not only test platelet contractility in response to antiplatelet agents, but also distinguish clot mechanics for health individuals from those with VWD. In short, the microfluidic systems of contractility measurement have demonstrated great potential in identifying platelet function disorders and giving additional anti-thrombotic therapeutic instruction.

### Highly Integrated Microfluidic Systems for Antiplatelet Drug Screening

To enable efficient and accurate antiplatelet drug screening from a large library in the presence of hemodynamic microenvironment, the microsystem should include the following characteristics: 1) cheap and easy-to-use with small volume requirement of blood samples; 2) Simple and user-friendly operation; 3) accurate and rapid testing; 4) high-throughput to test a large number of drug candidates (Meagher et al., 2008; Gubala et al., 2012; Myers et al., 2017; Berry et al., 2021). Hereby we reviewed several integrated and multiplexed microfluidic systems that meet these requirements, demonstrating great potentials towards rapid, automated and high-throughput antiplatelet drug screening.

Jose et al. (2016) designed an automated microfluidic device consisting of a PDMS layer with microchannels and a cyclic olefin polymer base printed with microcontact dot arrays (Figure 1E). The device achieves self-powered vacuum-driven flow by exposing the pre-degassed PDMS in the air. Here, dot array occupancy indicates platelet adhesion and the system has been used to screen GPIIb/IIIa and P2Y_12_ antagonists. Moreover, Berry et al. (2021) designed an occlusive thrombosis-on-a-chip model that incorporates two branching channels—a chaotic mixer for testing the EDRA quenching effects on platelet activation, and a collagen/tissue factor-coated channel for coagulation evaluation (Figure 1F). This model is simple and robust to measure occlusion time and can be utilized to screen potent antiplatelet drugs that inhibits channel occlusion and presumably blood vessel occlusion. Similarly, Szydzik et al. (2019) designed a novel active micropump mixer consisting of a pneumatic actuation chamber and a flow chamber separated by a thin diaphragm (Figure 1G). The micropump mixer enabled integration of sample preparation, drug incubation, blood mixing, and thrombus quantification on a single chip for antiplatelet drug screening.

Furthermore, Jongen et al. (2020) combined droplet microfluidics with flow cytometry for high-throughput single platelet function analysis (Figure 1H). The device incorporated four individual inlets to infuse platelets, agonist/antagonist solution and fluoro-oil, which eventually encountered at a common junction where analytes were encapsulated within the fluoro-oil droplets. Standard flow cytometry was then used to monitor droplet retrieved platelets’ response to convulxin—the agonist to platelet receptor glycoprotein VI. Besides, Hao *et al.* developed a platelet detection microfluidics that integrates chemotherapeutic agents, tumor cells, endothelial cells and the flow rates to predict platelet responsiveness from cancer patients before or during chemotherapy (Hao et al., 2021). The microsystem contained a drug concentration generator, cancer cell culture chips, and three-dimensional circular microchannels lined with confluent endothelial layers. Taken together, these highly integrated microdevices exhibit great potentials for scalable point-of-care application.

## Conclusion

Platelet mechanobiology inspired microfluidics are emerging technologies for rapid, robust, high-throughput thrombotic disease diagnosis and antiplatelet drug screening. Compared with the existing commercial platelet function tests, these microsystems are inexpensive and miniaturized, require small sample volume and have short processing time. As the manufacturing industry is rapidly advancing with respect to design standardization, operating procedure, analytical integration, we foresee that the microfluidic devices will evolve as not only cost-effective alternatives for basic platelet biology and anti-thrombotic pharmaceutical research, but also point-of-care and telehealth microdevices in cardiovascular patient management.
